# Optimizing Lactoferrin Isolation for Functional and Structural Integrity: A Molecular Insight

**DOI:** 10.3390/molecules31030454

**Published:** 2026-01-28

**Authors:** Ahmet Alperen Canbolat, Nur Hasret İstekli, Kadir Yılmaz, Mikhael Bechelany, Sercan Karav

**Affiliations:** 1 Department of Molecular Biology and Genetics, Çanakkale Onsekiz Mart University, 17100 Çanakkale, Türkiye; ahmetalperencanbolat@stu.comu.edu.tr (A.A.C.); hasretisteklinur@gmail.com (N.H.İ.); 2 Department of Chemical Engineering, Çanakkale Onsekiz Mart University, 17100 Çanakkale, Türkiye; kyilmaz@comu.edu.tr; 3 Institut Européen des Membranes (IEM), UMR 5635, University Montpellier, ENSCM, CNRS, F-34095 Montpellier, France; mikhael.bechelany@umontpellier.fr

**Keywords:** lactoferrin, isolation, affinity chromatography, colostrum, bioavailability

## Abstract

Lactoferrin (Lf) occurs predominantly within milk, coexisting with measurable levels across different glandular products and body fluids. Lf exhibits variation in relative molecular mass, influenced by its biological source and glycosylation profile; nevertheless, it is a close to 80 kDa glycoprotein. Provided that its bioactive structure is preserved, Lf performs a spectrum of physiological roles, comprising antioxidant, antifungal, antiviral, antiapoptotic, and antimicrobial actions. To sustain its bioactivity after isolation and ensure its effectiveness in subsequent applications, optimal conditions must be established throughout the optimization protocol, since inadequate optimization of parameters such as pH, temperature, ion balance, and protease activity may lead to aggregation, denaturation, and deterioration in functional regions, including the iron-binding domains. This review offers a comprehensive framework that associates isolation methodologies with structural integrity, preservation of iron-binding domains, and antimicrobial performance. Ion-exchange, affinity-based, and membrane-based approaches are systematically evaluated from analytical and functional perspectives, thereby yielding a synthesis that facilitates procedure selection and optimization for Lf isolation. In addition, the objectives of analytical characterization techniques implemented following isolation and the broadening scope of biotechnological applications of Lf are outlined.

## 1. Introduction

Lactoferrin (Lf) is a ~703 amino acid glycoprotein, notable for its relatively low proportions of methionine (~0.6%), histidine (~1.3%), and tryptophan (~1.5%), contrasted with its higher proportions of glycine (~7%), leucine (~9%), and alanine (~10%) [1,2]. Lf, which is classified within the transferrin family, is also referred to as lactotransferrin and is a family member with ~80 kDa. Lf exhibits different molecular weights depending on its source, appearing as 87–91 kDa in neutrophils and 83–87 kDa in bovine mammary gland, decreasing to approximately 77 kDa after deglycosylation [3]. Lf is an abundant iron-binding and major glycosylated (N-linked glycosylation) protein which is found in neutrophils within the bloodstream and epithelial secretions, namely saliva, mucus, and tears [4,5]. Since Lf is found among whey proteins, the amount within milk is related to the total protein level and this level may vary depending on the lactation period, SCC (somatic cell count) or other physiological factors [6,7,8]. Lf concentration is positively correlated with lactation stage; cows that have given birth more than once (multiparous cows) show higher Lf levels compared to cows that are in their first lactation [8]. The first stage of the lactation period, which corresponds to colostrum produced within the first three days after calving, contains about 5.75 mg/mL of Lf in bovine colostrum, and as the lactation period progresses and milk transitions into mature milk, Lf concentration decreases [9,10]. Mastitis refers to the inflammation of the udder triggered by bacterial infection, is associated with increased Lf concentration and elevated somatic cell count (SCC) in milk; while normal bovine milk have average Lf level of 0.35 mg/mL, bovine with mastitis show higher levels ranging from 0.318 to 2.270 mg/mL, highlighting Lf’s antibacterial role in the immune response [11,12,13,14].

Lf, a bioactive compound derived from milk, is important for lifelong health due to its effectiveness in infant nutrition, promotion of intestinal homeostasis, and bioavailability effects [15,16]. It possesses various bioactive functions, among which are antimicrobial, antioxidant, anti-apoptotic, antifungal, anti-inflammatory properties, immunity regulating, inhabitation of lipid peroxidation and cancer prevention [6,15,17,18]. Besides its known properties, the antiviral and immunomodulatory effects of Lf have been explored in various studies, such as its use in detecting rotavirus via immunoassays and promoting tendon healing through surface-enabled applications [19,20]. The high iron-binding affinity (Kd ≈ 10^−20^ M) of Lf, along with its low iron saturation, has defined it as a ‘bacteriostatic agent’ [21]. Lf an iron-binding glycoprotein, chelates ferric ions within the Lf structure, and this chelating ability can also explain the antimicrobial effect of Lf [18]. In other words, bacteria need iron in many biochemical functions, host defense mechanisms, and other intracellular activities, so iron starvation carries a risk of death, but these functions could be usable in combating microbial infection [22,23]. It also has the ability to chelate other transition metals, but it varies depending on ions and proteins present, and this is explained as broad-spectrum chelating. Lf strongly binds cobalt (III), copper (II), iron (III), chromium (III), and manganese (III) ions (with bicarbonate), while it weakly interacts with several other metal ions, with manganese (II) being oxidized to manganese (III) when bound [24].

With its unique properties, Lf has become isolable and purifiable so that it can meet the demand in its areas of application. In this context, various new techniques have been and continue to be developed depending on the desired Lf structure; remarkably, in recent decades, Lf manufacturing has climbed from below 80 tons to above 300 tons [25]. During the purification of Lf, it is important to ensure that the glycoprotein is obtained without losing its biological activity, as pure Lf can later be incorporated into the formulation of enriched foods, pharmaceuticals, cosmetics, and packaging materials due to its biological functionality [26]. Lf N-linked glycosylation serves an essential function in the immunological protection system in response to a range of microbial challenges. Therefore, isolation studies pay close attention to preserving the glycan structure. Lf’s glycosylation profile is heterogeneous across sources and taxa, which not only makes Lf unique but also shapes the purification techniques accordingly [27]. Commercially, Lf is currently isolated from bovine and similar daily sources, owing to the ease of its procurement and relatively low cost, and in order to increase the concentration of Lf obtained from such sources, efforts are ongoing to design transgenic animals along with the development of related technical approaches [28]. The objective of this review is to systematically analyze isolation methodologies, including ion-exchange chromatography, affinity-based systems, and membrane-based technologies, not only in relation to attaining superior purity and yield, but also concerning their capacity to sustain molecular integrity and functional performance. In addition, the approaches used in Lf isolation experiments from diverse sources, the importance of optimization, the methods employed, and the key factors must be considered are presented. Furthermore, the functional applications of Lf obtained under conditions that preserve its properties are reported, and new approaches are elaborated with a range of examples. While many existing reviews discuss the biological functions of Lf and purification techniques in distinct sections, this review integrates isolation strategies with biological outcomes, particularly structural stability, integrity of the iron-binding domains, and antimicrobial activity, within a single cohesive framework.

## 2. Molecular and Structural Properties of Lactoferrin

### 2.1. Protein Structure

The presence of Lf exclusively in mammals makes it a species-specific structure, while Lf-like glycoproteins are found in other species; it has been proposed that Lf originated from a duplication of the transferrin (*Tf*) gene and later diversified through differences in amino acid sequences, and although similarity rates among the amino acid sequences of nine mammalian species range between 65% and 100% the highest similarity was observed in the Lf’s of cow, buffalo, goat, and sheep [29]. The primary structure of Lf comprises a linear arrangement of roughly 689 monomers; specific positions within this sequence form functional regions responsible for glycosylation, iron binding, and antimicrobial activity [30]. Featuring α-helices accounting for approximately 33–34% and β-strands 17–18%, these structural motifs compose the secondary structure of Lf. In terms of tertiary stabilization, bovine lactoferrin (bLf) is supported by approximately 17 disulfide bonds. At a resolution of 2.8 Å, the tertiary conformation of human lactoferrin (hLf) and bLf has been elucidated and defined [21].

X-ray crystallographic analysis demonstrated that the tertiary architecture of Lf adopts a bilobal structure, consisting of the amino-terminal (N) and carboxyl-terminal (C) lobes. Both domains are connected by a short α-helix through hydrophobic interactions, contributing to its unique three-dimensional conformation. Each lobe shares about 33–41% sequence identity, and both contain α/β folds—designated as C1, C2, or N1, N2—which harbor the iron-interacting regions. Amino acid residues 345–431 and 593–676 are composed of C1 in the C-lobe, and residues 432–592 make up C2. In the N-lobe, polypeptide segments 1–90 and 251–333 comprise the N1 sub-lobe, while segments 91–250 form the N2 sub-lobe. A small 3-turn helix formed by amino acid residues 334–344 bridges N-terminal and C-terminal domains while simultaneously adding flexibility to the structure in binding and releasing iron [5,31]. Although the two metal-binding motifs located in the N- and C-lobes of Lf differ in their metal affinities, they share a similar structure; each site contains 2 Tyr (Tyr 93 and Tyr 193), 1 His (His 254), and 1 Asp (Asp 61), through which the metal ion is coordinated. Upon the addition of a carbonate anion, a bridge is formed within the binding site, stabilizing the structure and completing the octahedral geometry [32]. The factor inducing a conformational change of the N-lobe is the rigid-body reorientation of about 54° undertaken by N2 in relation to N1. These structural rearrangements give rise to a salt bridge, which enables either ligand binding originating in the C-lobe or signal transmission extending toward the N-lobe; consequently, the orientation of both lobes is altered [33].

Due to its reversible binding property, the two forms, the apo version alongside the holo version, have the capacity to coexist within the structure. In this context, Lf in its holo state represents the iron-saturated conformation and displays greater susceptibility to proteolysis compared to the open-structured apo-Lf; this difference is attributed to the conformational arrangement of the lobes and to the fact that the apo form, lacking iron, adopts a more open molecular structure [34]. Anion binding is required to avoid a net positive charge in the metal-binding site that could hinder coordination; thus, metal and anion binding occur synergistically. To illustrate, under the conditions of two carbonate ions (CO_3_^2−^), two ferric ions (Fe^3+^) are coordinated to Lf via covalent linkage. Although Lf possesses pronounced specificity for Fe^3+^, it can also interact with other divalent or trivalent transition metal ions [29].

### 2.2. Stability and Functionality

The iron release triggered by pH, as examined through pH kinetics, proceeds as follows: a decrease in pH weakens iron coordination, interacts with existing positive charges, and causes the lobes to release iron into the environment. Attributable to the differences between the C- and N- lobes across species, the pH-dependent process of iron association and its consequent liberation varies. For instance, in bLf, the C-lobe begins to liberate iron at pH 5.5, whereas the N-lobe starts releasing ferric ions at pH 6.5. In contrast, hLf behaves differently; its N-lobe begins metal ions release at pH 5.0, whereas the C-lobe does not release ferric ions until around pH 3.5. In inflammatory conditions where the pH drops to 4.52, the capacity of Lf to sequester iron at low pH is of high significance, as it negatively affects the metabolic activity of bacteria. The divergent interaction patterns characterized by the N- and C-lobes, which constitute the iron-binding sites within the structure of Lf, highlight that optimization of these molecular attributes is critical for the selection of rational isolation strategies and for achieving efficient outcomes. Failure to preserve this structural heterogeneity throughout the isolation process gives rise to structural perturbations in the iron-binding regions, which consequently result in attenuated antibacterial activity and weakened effectiveness of other functional properties of the recovered protein [35]. Findings from a study reveal that Lf is characterized by stable and active regions that are resistant to degradation at acidic pH but are easily denatured by heat treatment near neutral pH [36]. Lf, despite varying among species, generally features a high isoelectric point (pI). Notably, the pI of hLf is 9.5, while that of bLf is 9.4. This difference arises from the amino acid composition, chiefly arginine, which imparts a positive charge to Lf and accounts for its basic nature [37]. Strong acids or bases, extreme temperatures (either high or low), and environments rich in inorganic or organic salts are among the primary factors that cause protein denaturation. These conditions can profoundly affect not only the biological activity of proteins but also their conformation, three-dimensional structure (including secondary, tertiary, and quaternary levels), the stabilizing forces (such as hydrogen and disulfide bonds), and their functional properties, including iron-binding capacity and antibacterial activity [5]. In its iron-saturated form, against protease-mediated breakdown by trypsin and chymotrypsin, Lf remains stable. Subsequent studies have revealed that the resistance exhibited in the intestines of breast milk-fed infants against pancreatic enzymes and pepsin is explained by the presence of full-length Lf in their feces. This highlights the significant proteolytic resistance of Lf to its physiological roles [21]. Research indicates that the bioactivity of Lf increases after hydrolysis with pepsin; this has been attributed to lactoferricin, a bioactive peptide released during enzymatic cleavage. Lactoferricin covers the 18–42 residue sequence positioned within the N-terminal domain of bLf. Within this 25-amino acid sequence, 8 amino acids are basic characteristically, and 6 of them are found within the N-terminal α-helix structure. Owing to this basic (positively charged) nature, lactoferricin promotes strong electrostatic interactions with negatively charged pathogenic cellular membranes, thereby potentiating its antimicrobial activity. Structural damage, misfolding, or chemical modification of Lf throughout isolation undermines the integrity of the parent protein, consequently impeding the functional generation of lactoferricin. In contrast, preservation of the native parent structure facilitates the generation of structurally intact lactoferricin, which is correlated with augmented antimicrobial activity. This increase in biological activity can also be explained by the antimicrobial effect of lactoferricin, which is associated with its ability to form ion channels in the cell membrane due to its α-helical, cationic, and amphipathic structures [38]. Asparagine moieties located in the C- and N-lobes of Lf’s primary structure constitute N-glycosylation loci, while the abundance and placement of cysteine moieties are instrumental in the construction of disulfide bridges. Interspecies differences in these glycosylation sites, referred to as glycovariations, can influence Lf’s sensitivity to proteolytic degradation and heat-induced unfolding. Glycosylation does not interfere with iron or other ligand binding; in fact, the carbohydrate chains present in the structure may enhance resistance to proteolysis. Furthermore, glycosylation performed via the Maillard reaction (a non-enzymatic glycosylation process) has been reported to improve the thermal stability, emulsifying capacity, foaming ability, and antioxidant activity, while also reducing surface hydrophobicity and increasing total sulfhydryl content of Lf. The regions suitable for N-glycosylation vary among species, notably with mouse Lf possesses one loci (Asn476), hLf possesses three loci (Asn137, Asn478, and Asn623), and bLf possesses four loci (Asn233, Asn368, Asn476, and Asn545) [39,40].

## 3. Isolation Methodologies: Traditional and Molecular Approaches

In the process of Lf isolation, the Lf level of the origin matrix, together with the array of purification approaches employed, are fundamental considerations. Conventional methods such as affinity chromatography and ion-exchange chromatography are optimized based on the characteristics of the intended protein, and the implementation of distinct resins can enhance the purity of the final product. When these methods are combined with pre- or auxiliary procedures that elevate protein concentration (e.g., ultrafiltration), both time and cost efficiency are improved, as demonstrated by the validation methods employed (see Table 1).

### 3.1. Pre-Treatment of Samples

The pre-treatment of raw milk or colostrum samples in advance of isolation is a critical step in the successful isolation of Lf, as it ensures removal of interfering components while preserving protein stability. The process generally begins with centrifugation to separate fat and obtain skimmed milk, which minimizes lipid contamination during subsequent purification stages. Casein proteins, which constitute the bulk of milk protein, are then removed by acid precipitation at their isoelectric point (pH~4.6), yielding a clarified whey fraction enriched in soluble proteins, including Lf. Additional clarification steps, such as microfiltration or low-speed centrifugation, are often employed to eliminate residual particles and cellular debris. Stabilization of the sample is achieved by adjusting buffer conditions to maintain optimal pH and ionic strength, while protease inhibitors may be added to prevent enzymatic degradation. Temperature control at 4–10 °C maintains the structural and functional integrity of Lf before downstream chromatographic purification. The sequence of pre-treatment measures creates the basis for isolating Lf in large amounts and with high purity, while preserving its bioactivity [41,42].

### 3.2. Traditional Methods

#### 3.2.1. Ion-Exchange Chromatography

Among the prominent purification methods, ion-exchange chromatography is widely recognized as the standard for the purification of Lf from initial raw materials like skim milk, whey and colostrum because of the relatively high isoelectric point of Lf, allowing selective binding to cation-exchange resins at acidic pH [45,46]. Both batch and continuous feeding are possible, and gradient elution using increased salt concentrations can yield a product of very high purity. The use of expanded bed adsorption and other advanced resin systems has improved throughput with fewer processing steps [47]. Recent efforts also focused on the simultaneous capture of Lf together with other functional proteins, e.g., lactoperoxidase, from raw or lightly processed milk [45].

Bovine whey was used as the source of Lf. Fat globules in conjunction with particles were eliminated via centrifugation of the raw material at 3000× *g* for 20 min under refrigerated conditions. Through regulating the acidity using 1 M HCl, caseins were aggregated until it reached 4.6 and holding the suspension at 4 °C for 30 min, and centrifuging at 4000× *g* for 25 min. This clarified liquid fraction was filtered using a 0.45 μm membrane and subsequently modulated at pH 6.2 employing 1 M NaOH. Reduction of ionic strength occured by dilution with an equilibration buffer to achieve a conductivity ≤ 3 mS/cm, as lower salt concentrations enhance protein binding to cation exchangers.

Lf was purified using a strong cation exchange resin (SP-Sepharose Fast Flow; Cytiva). The column (10–20 cm bed height, aspect ratio 1:10–1:15) was packed and equilibrated with 10 column volumes (CV) of equilibration buffer consisting of 50 mM sodium phosphate, pH 6.2, containing 10–50 mM NaCl. The sample was loaded using a linear velocity of 50–100 cm/h, not exceeding 80% of the resin’s dynamic binding capacity (20–40 mg protein/mL resin). Following sample application, the resin bed received a rinse through 8–10 CV of pre-conditioning aqueous medium until baseline absorbance at 280 nm was reached. Bound Lf was collected using a progressive salt ramp over a span of 0 to 1 M NaCl in an equilibration solution over 15–20 CV. In agreement with prior reports, Lf typically eluted between 0.35 and 0.55 M NaCl. Protein-exhibiting peaks were identified by spectrophotometric measurement at 280 nm, and the portions (2–5 mL) were gathered. Elution fractions were analyzed by 12% SDS-PAGE under reducing conditions, and those containing predominantly Lf (~80 kDa band) were pooled.

**Figure 1:**
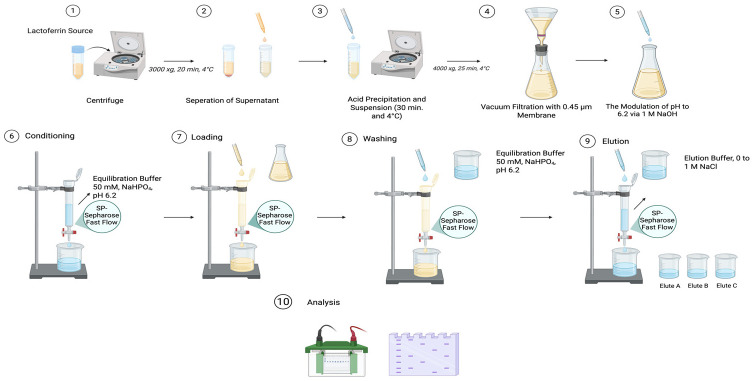
A basic illustration of ion-exchange chromatography. For Lf ion-exchange purification, lactoferrin sources were clarified by centrifugation (3000× *g*, 20 min, 4 °C) to remove fat globules and particulates. Caseins were precipitated by acidification to pH 4.6 with 1 M HCl, incubated at 4 °C for 30 min, and centrifuged (4000× *g*, 25 min). The supernatant was filtered (0.45 μm) and adjusted to pH 6.2 with 1 M NaOH. Conductivity was reduced (≤3 mS/cm) by dilution with equilibration buffer (50 mM sodium phosphate, pH 6.2, 10–50 mM NaCl). Lf was captured using a strong cation exchange column (SP-Sepharose FF; Cytiva), equilibrated with 10 CV of the same buffer. The sample was loaded at 50–100 cm/h (≤80% resin capacity), washed (8–10 CV), and eluted with a 0–1 M NaCl linear gradient over 15–20 CV. Lf typically eluted at 0.35–0.55 M NaCl. Fractions showing an ~80 kDa band on 12% SDS-PAGE were pooled. The pooled Lf was buffer-exchanged into 10 mM sodium phosphate, pH 7.0, using ultrafiltration (10–30 kDa MWCO) to concentrate to 10–20 mg/mL. Protein concentration was determined by the Bradford assay (BSA standard, Sigma, Oakville, ON, Canada, 2017) [46]. Aliquots were snap-frozen in liquid N_2_ and stored at −80 °C. Purity (>90%) was confirmed by SDS-PAGE and OD_280_ measurements.

Pooled fractions were conditioned in 10 mM sodium phosphate, pH 7.0, using ultrafiltration (10–30 kDa MWCO). Ultrafiltration was selected over dialysis for improved efficiency and reduced contamination risk. Analyte was enriched achieving 10–20 mg/mL followed by quantification employing the Bradford’s protein determination using bovine serum albumin (BSA) as the calibrator. Instantaneously frozen aliquots within liquid nitrogen were preserved under –80 °C. Purity, exceeding 90%, was characterized through SDS-PAGE, and the concentration was confirmed by optical density measurement at 280 nm (Figure 1).

#### 3.2.2. Affinity Chromatography

Affinity chromatography is a separation method that is highly selective and efficient for specific protein purification, exploiting the specific binding recognition established between a target entity and a binding partner fixed onto a solid support. Upon movement of a complex mixture of intended proteins across the chromatographic matrix, particulates exhibiting selective interaction attach tightly to the recognition element, whereas the remaining components are removed during rinsing. By altering hydrogen ion concentration, electrolyte level, or incorporating an antagonistic effector, the captured molecule of interest can be desorbed, resulting in a highly purified product. Studies leveraging the binding moiety-recognition potential of Lf have also been documented. One such approach involves the use of ligands with high affinity, such as DNA, which are immobilized onto the column; in this way, Lf is selectively retained and thereafter desorbed. In contrast, in purifications implemented through metal-affinity chromatography (IMAC), the rationale is founded on the iron-association potential of the polypeptide. In this case, the affinity of the polypeptide is dependent on its side chains and the particular metal ion employed [43,44].

The Lf source is collected and prepared for extraction. The sample is sedimented at 4000× *g* for 30 min under 4 °C to separate the adipose fraction and partially remove major milk protein aggregates, which interfere with protein isolation. Following gravitational separation, the hydrophilic supernatant, located amid the cream stratum and the casein deposit, is carefully recovered. To eliminate residual caseins, the acidity level in the liquid fraction is modified to 4.6 through the addition of HCl. Alternatively, caseins are precipitated through incorporation of CaCl_2_, reaching a total level of 60 mM, adjusted to pH 4.6. Both preparations are subjected to centrifugation under the same conditions, and the resulting supernatants are collected. This centrifugation step is repeated several times if necessary to improve casein removal and minimize interfering components. The clarified, acid-precipitated supernatant is concentrated twenty-fold using a centrifugal filter unit with a 50 kDa size exclusion limit (Amicon, Millipore, Danvers, MA, USA). The enriched fraction is resuspended in operational buffer (0.1 M Tris-HCl, pH 8.0), optionally supplemented with 0.05% polysorbate−20 and 50 mM NaCl and gently mixed for 30 min prior to chromatography.

**Figure 2:**
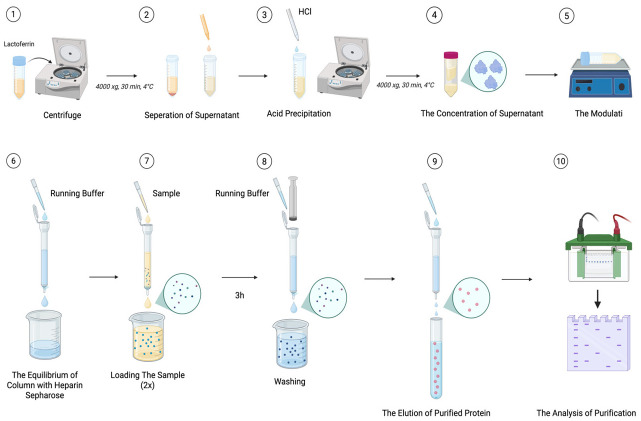
A basic illustration of affinity chromatography. For Lf affinity purification, milk fractions were centrifuged at 4000× *g* under 4 °C to remove fat and aggregates, followed by casein precipitation using either acidification to pH 4.6 or addition of CaCl_2_ (60 mM, pH 4.6). The clarified supernatant was collected, concentrated twenty-fold with a 50 kDa size exclusion filter, and resuspended in 0.1 M Tris-HCl buffer (pH 8.0) supplemented with optional surfactant and NaCl. The prepared fraction was applied to a heparin–Sepharose column, with the flow-through reapplied to maximize binding, and incubated for 3 h at room temperature. After washing away nonspecific proteins, Lf was eluted stepwise using a NaCl gradient (0.1–1.0 M). Collected fractions were analyzed by SDS-PAGE, and those enriched in Lf were pooled, dialyzed, and quantified by the Bradford assay.

For affinity purification, heparin–Sepharose beads are manually packed into a polypropylene column and equilibrated with elution medium. Following preparation, the fraction is loaded onto the resin, with the flow-through being reapplied twice to enhance Lf binding. Binding is allowed to proceed for 3 h at room temperature. Nonspecifically bound proteins are removed by washing with running buffer, and Lf is eluted stepwise in the presence of NaCl gradients spanning 0.1–1.0 M. Collected fractions are analyzed by 12% SDS-PAGE to evaluate protein composition. Fractions enriched in Lf and showing minimal contamination are pooled and dialyzed against deionized water using Spectra/Por^®^ dialysis tubing, (Rancho Dominguez, CA, USA). (6–8 kDa size limit). Protein quantities are evaluated using the Bradford procedure, with BSA as a reference (Figure 2).

### 3.3. Ultrafiltration and Membrane Technologies

Membrane separation technologies, principally ultrafiltration (UF), microfiltration (MF), and diafiltration (DF), have become established as fundamental unit operations for the primary recovery and concentration of Lf from complex dairy streams [48,49]. Their operational efficacy, scalability, and relatively low cost underpin their widespread adoption in both pilot and industrial-scale bioprocessing.

**Figure 3:**
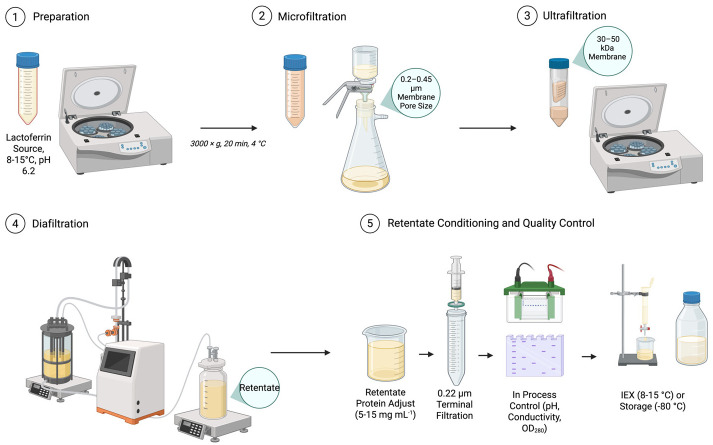
A schematic of ultrafiltration and membrane technologies. Membrane separation technologies—microfiltration (MF), ultrafiltration (UF), and diafiltration (DF)—form the core of lactoferrin (Lf) recovery from dairy streams, enabling efficient clarification, concentration, and conditioning prior to ion-exchange chromatography (IEX). MF (0.1–1.4 µm) removes fat globules, casein micelles, and particulates to protect downstream UF membranes. UF using 30–50 kDa MWCO membranes retains Lf (~80 kDa) while permeating lactose, minerals, and small proteins, concentrating Lf 5–10× at 8–15 °C and pH 6.0–6.2. Subsequent DF with 25–50 mM phosphate buffer (pH 6.2) for 2–6 diavolumes lowers conductivity (≤3 mS cm^−1^), optimizing conditions for cation-exchange binding. The conditioned retentate (5–15 mg mL^−1^ protein) may undergo 0.22 µm filtration for bioburden control, with in-process monitoring of pH, conductivity, OD_280_, and SDS-PAGE profiles. Lf retentates are loaded immediately onto IEX columns or stored briefly at 2–8 °C, providing a robust, scalable platform for high-purity lactoferrin isolation.

Microfiltration, typically employing membranes with pore sizes of 0.1 to 1.4 µm, serves as a critical pre-treatment step. Its primary function is the removal of colloidal particulates, residual fat globules, and casein micelles, while simultaneously reducing the bioburden of the feed material, thereby protecting downstream UF membranes from fouling and ensuring process hygiene [49]. Following clarification, ultrafiltration performs the initial purification and volume reduction. By utilizing membranes with a molecular weight cut-off (MWCO) in the range of 10–100 kDa, UF effectively segregates the high molecular weight Lf (approximately 80 kDa) from lower molecular weight solutes, including lactose, minerals, and smaller whey proteins. This process concentrates Lf in the retentate fraction, significantly increasing its specific activity and preparing it for subsequent polishing steps. Industrially, the integration of these membrane processes with ion-exchange chromatography (IEX) represents a robust and scalable production platform. The conditioned UF retentate, often subjected to diafiltration to lower ionic strength, is ideally suited for high-capacity binding to IEX resins, enabling high-purity Lf isolation [17]. Current scholarly reviews highlight a growing research focus on synergizing conventional UF with advanced chromatographic techniques to further enhance process efficiency and output purity. This includes the integration with methods that exploit enlarged phase interactions, immobilized metal affinity chromatography (IMAC), and hybrid matrix membrane fractionation, all aimed at maximizing yield and purity in a single, streamlined operation [25]. Furthermore, the scientific literature points to the significant promise of emerging, high-selectivity isolation systems. Novel approaches utilizing magnetic beads functionalized with Lf-specific ligands and nanoparticle-based capture systems are under active investigation. These technologies offer the potential for highly selective binding and efficient separation from crude mixtures, representing a promising frontier for future advancements in the cost-effective and high-resolution purification of Lf [5]. Ultrafiltration and membrane conditioning proceed on clarified dairy feed as follows. Skim milk or sweet whey is pre-cooled (8–15 °C), adjusted to pH 6.0–6.2, and clarified by centrifugation (3000× *g*, 20 min, 4 °C) to remove fat globules and coarse particulates. The supernatant may pass through microfiltration (0.2–0.45 µm; TMP 0.5–1.5 bar; cross-flow 3–6 m s^−1^) to reduce casein fines and bioburden; the permeate serves as the UF feed. Ultrafiltration uses a 30–50 kDa MWCO membrane operated at 1–3 bar TMP and 1–4 m s^−1^ cross-flow while maintaining 8–15 °C and pH 6.0–6.2 to minimize aggregation. The retentate concentrates to a 5–10× volumetric concentration factor with continuous flux monitoring; concentration halts if viscosity-driven pressure rise occurs. Without changing hold-up, diafiltration then employs a low-ionic-strength buffer (25–50 mM sodium phosphate, pH 6.2) for 2–6 diavolumes until conductivity reaches ≤3 mS cm^−1^, which supports high-capacity binding on cation-exchange media. Retentate protein adjusts to 5–15 mg mL^−1^ for column loading; where bioburden control is critical, a 0.22 µm terminal filtration is applied. In-process control tracks pH and conductivity after each diavolume, OD_280_ of permeates to verify minimal Lf passage, and periodic 12% SDS-PAGE of retentate to confirm the protein profile. Conditioned retentates transfer immediately to IEX or hold at 2–8 °C for ≤24 h; for longer storage, aliquots snap-freeze at −80 °C. Between runs, membranes rinse with water, undergo alkaline cleaning, acid rinse, thorough water flush and sanitation prior to reuse (Figure 3).

### 3.4. Comparative Evaluation of Lactoferrin Isolation Strategies

Ion-exchange chromatography, affinity chromatography, and membrane-based separation offer distinct advantages and limitations in lactoferrin (Lf) isolation, respectively. Ion-exchange chromatography exploits the high isoelectric point of Lf and provides high purity and scalability; however, it requires precise control of pH and ionic strength and is sensitive to fouling when applied directly to complex matrices [45]. Affinity chromatography offers enhanced selectivity and structural preservation, making it particularly suitable for analytical and glycan-focused applications, but its high resin cost and limited scalability restrict industrial implementation [43]. Membrane-based techniques, including microfiltration and ultrafiltration, do not replace chromatographic purification but function as critical pre-conditioning steps that clarify, concentrate, and stabilize the feed stream. By removing fat globules, casein micelles, and particulates, microfiltration significantly reduces column fouling, enhances binding efficiency in subsequent ion-exchange steps, and improves overall process robustness [48]. Consequently, membrane filtration and ion-exchange chromatography should be viewed as complementary rather than redundant technologies within an integrated lactoferrin isolation workflow.

## 4. Risks of Structural and Functional Loss

### 4.1. Denaturation Mechanisms and Critical Factors

The preservation of Lf’s structural integrity and biological function during isolation, processing and storage operations represents a significant challenge in the commercialization of this protein. Comprehending the unfolding mechanisms is essential for implementing appropriate mitigation strategies to maintain product quality and bioactivity during all stages of production and use.

#### 4.1.1. Thermal Denaturation

Thermal treatment poses a critical risk for the denaturation of Lf in industrial processes, with heat treatment having a more adverse effect on the tertiary structure of Lf than on its secondary structure. Recent studies have shown that Lf has different thermal denaturation properties in various species. BLf exhibits an endothermic peak at 65.71 °C, whereas hLf demonstrates greater thermal stability, denaturing at 90.05 °C [50]. Temperatures for denaturation are dependent on the processing environment since Lf maintains its structure during pasteurization at 60–75 °C but begins to lose its functionality at elevated temperatures [51].

The iron saturation level has a considerable impact on thermal stability, with samples of Lf having maximum physical and conformational integrity at a saturation level of 40%. In contrast, an iron saturation level of below 15% presents susceptibility to brief thermal exposure, reaching 95 °C with a duration of 15 s [52]. This has significant implications for the optimization of process parameters, as higher levels of iron provide defense against heat-induced denaturation [53]. The improved thermal stability of iron-saturated Lf is attributed to the protective nature of iron binding to the tertiary structure of the protein, thus helping to preserve conformational integrity under thermal stress.

Industrial spray drying presents specific challenges that impact the thermal stability of Lf, primarily due to the interaction of high temperatures and the rapid removal of moisture. However, innovative thermal stabilization methods have been developed, including one involving sorption in mesoporous silica. This method has the potential to considerably enhance thermal stability by creating a protective microenvironment that preserves the structural integrity of proteins during high-temperature processing [54].

#### 4.1.2. pH-Induced Denaturation

Lf has a pH-dependent stability profile with specific regions of susceptibility that must be carefully controlled during processing and formulation. It has its function preserved within a pH range of 6 to 9, but the protein completely loses antimicrobial function when treated at a pH of 3 to 5 [51]. This property represents a significant challenge to oral delivery, as the acidic milieu of gastric fluid, characterized by a pH range of 1.5 to 3.5, can quickly denature the protein’s structural integrity and compromise its biological function.

At pH 5.0, a significant destabilization of structural integrity is evident, with bLf showing a decrease of 21.78 °C in the denaturation temperature compared to the neutral state [50]. This pH changes compromises both secondary and tertiary structural elements, thus affecting the kinetics and iron-binding capacity, causing enhanced sensitivity to proteolytic degradation and weakening of antimicrobial and antioxidant activities. The irreversible effects related to extensive pH-induced unfolding underscore the importance of ensuring strict pH control at each step of the formulation.

Industrial processing maintains the pH in a defined range of 6.0 to 8.0 to prevent denaturation and enable proper separation. Recent research on advanced pH-sensitive drug delivery systems has addressed the issue of gastric stability by designing complex formulations capable of preserving lactoferrin in acidic environments while providing controlled release through increased alkalinity [55].

#### 4.1.3. Chemical and Oxidative Denaturation

The structural configuration of cysteine residues in interconnected disulfide bonds defines Lf and offers essential assistance in its compactly folded and stable conformation. However, these bonds can be selectively cleaved in oxidative environments, causing protein unfolding, together with a consequent impairment of its structural stability. The following oxidation mechanisms remain clinically pertinent: methionine oxidation causes overall folding abnormalities; tryptophan oxidation causes disruption of the coordination environment of ferric iron and oxidation of electrophilic peptide residues through tyrosine reduces antimicrobial performance. Such oxidative injury can accrue steadily in standard storage environments or can be considerably heightened in extreme processing environments, thus emphasizing the pressing need for broad-spectrum antioxidant delivery protocols.

Hybrid systems comprising Lf and stabilizing enzymes can potentially boost oxidative resistance through biomimetic surface immobilization. This method concurrently retains both the tertiary structure of the protein and its catalytic activity [56]. Although iron coordination plays a crucial role in several physiological activities, a surplus of transition metal cations—particularly Cu^2+^, Mn^2+^, and Zn^2+^—poses serious risks. These cations can potentially compete for the ferric binding site, induce oxidative injury via Fenton-like reactions, promote intermolecular cross-linkage, and eventually accelerate protein turbidity and aggregation, compromising the functional bioavailability of the peptide.

### 4.2. Proteolytic Degradation Risks

Proteolytic degradation poses a substantial threat to Lf functionality, exerting pressure at various stages, including processing, storage, and physiological application. During gastrointestinal traversal, Lf encounters pronounced proteolytic risks, particularly in the acidic gastric milieu. Evidence indicates that unencapsulated Lf exhibits heightened susceptibility to gastric pepsin and subsequent trypsins compared to encapsulated dairy and nondairy vehicles [57]. Recent in vitro studies simulating the complete intestinal continuum have revealed that Lf degradation occurs in kinetically distinct phases. While gastric specimens release less than 20% intact protein, nearly all detectable Lf degrades in simulated colonic media when the polypeptide is appropriately protected in chitosan-based microparticles or nanolattice gels [58]. The contrast in release kinetics underscores the importance of designing target-oriented delivery vehicles to preserve the tertiary and quaternary structural integrity of Lf along the entire gastrointestinal pathway. Gastric digestion of free Lf results in a partial loss of in vitro antibacterial effectiveness against Listeria monocytogenes, with residual activity markedly below that of the native protein [59]. In contrast, Lf that has been appropriately encapsulated or ensconced within protective matrices displays considerably greater resistance to in vitro digestion, sustaining antibacterial activity, which underscores the pivotal role of formulation approaches in safeguarding the protein’s biological efficacy. Proteolytic cleavage initiates at predictable sites, commencing between the homologous N-lobe- and C-lobe segments, yielding fragments that, while potentially capable of partial iron chelation, generally exhibit attenuated functionality. Further proteolytic events and the distribution of cleavage sites are determined by the gastrointestinal proteolytic milieu, prevailing pH, and inclusion of stabilizing excipients or targeted delivery systems. Dairy raw materials are subject to protein breakdown by a variety of endogenous enzymes: plasmin predominates in the neutral pH range of 6.5–7.5, cathepsins operate in acidic environments, bacterial enzymes can arise from microbial contamination, and proteases from somatic cells are elevated in mastitis milk. Key prevention methods include selected thermal treatments to achieve broad protease inactivation, pH optimization that steers the matrix outside the precarious activity regions, supplementation with protease inhibitors—such as EDTA or more targeted peptides—and accelerated processing that truncates retention time in vulnerable zones. Prolonged storage, even under rigorously controlled parameters, can facilitate proteolytic degradation. These mechanisms include autocatalytic release at moderately elevated temperatures, survival of incomplete inactivation protocols, non-enzymatic cleavage under extended temperature exposure, and preferential attack of protein aggregates that form during storage. Effective storage tactics include using temperatures strictly below −20 °C for extended retention or 4 °C for short-term situations, maintaining a pH of 7.0–7.5, limit moisture below 5% in powdered forms, employing inert nitrogen or vacuum environments to avert oxidative degradation, and adopting UV-blocking packaging to exclude light that could catalyze oxidation or thermal instability.

## 5. Molecular Approaches

### 5.1. pH Optimization and Buffer Systems

The formulation of robust pH optimization protocols must proceed from a comprehensive grasp of the interdependencies among pH, Lf integrity and the distinct performance criteria demanded by disparate applications. Recent investigations have delineated narrow pH windows governing the stability of Lf-polysaccharide ensembles, revealing that each polysaccharide cohort elicits a discrete stability profile and therefore dictates a corresponding pH adjustment for peak protective efficacy [60]. The formation of Lf-polysaccharide complexes results in profound shifts in pH-sensitive stability. These covalent and ionic bond networks shield the Lf moiety against acid-induced unfolding, a protective effect that heightens thermal resistance, mitigates proteolytic susceptibility, and augments the bioavailability of the iron-binding glycoprotein in physiological matrices. Consequently, pH fine-tuning must be executed with consideration of the final formulation architecture and the designated delivery milieu, as the maximum stability, activity, and biocompatibility differ markedly among the polysaccharide constituents and the anticipated transport conditions. Targeted pH manipulation induces structural refinements in Lf, enhancing both its permanence and functional efficacy without compromising its native biological activities. Sophisticated formulation paradigms harness pH-sensitive matrices to safeguard Lf against gastric degradation and orchestrate the precise liberation of the protein in the intestinal milieu. Recent innovations in chitosan-centric microbead delivery systems have achieved colon-specific targeting. These microspheres incorporate pH-reactive polymers that retain their mechanical integrity under acidic conditions but swell and release their encapsulated Lf upon exposure to the neutral to alkaline conditions of the distal colon [55]. Conventional single-component buffering agents frequently fail to ensure stability across the entire formulation, processing, and shelf-life phases. This limitation has prompted the design of multi-component buffering environments that confer superior pH regulation. Such systems operate not only through proton-sponge action but also through coordinated metal chelation, antioxidant, and protein-cosolvent mechanisms. The latest generation of these biocompatible buffering formulations is specifically tailored to preserve Lf functional integrity while simultaneously augmenting its bioavailability in therapeutic and dietary applications, thereby translating basic stability into enhanced performance.

### 5.2. In Vitro Strategies for Stability Enhancement

Nanoencapsulation is one of the most effective technologies for shielding Lf against enzymatic degradation while preserving its bioactivity. Recent investigations have highlighted the impressive efficacy of chitosan nanoparticles, which enable Lf to exhibit markedly improved resistance to trypsin and pepsin, along with sustained antibacterial activity [57]. These chitosan formulations yield particles of 150–300 nm diameter, a polydispersity index indicating a narrow size distribution, and an encapsulation efficiency of 85–95% when the formulation parameters are optimized. Following proteolytic challenge, the bioencapsulated Lf retained over 90% of its antibacterial potency, providing compelling evidence of particle stability. Emerging methodologies for Lf stabilization feature the covalent coupling of the protein with plant-derived polyphenolic compounds, which afford complementary stabilization effects. Recent studies have shown that plant-derived recombinant human lactoferrin (rhLf) linked to a spectrum of polyphenol ligands yields stable covalent conjugates that exhibit improved physicochemical characteristics and a more favorable gastrointestinal profile [61]. These polyphenol-Lf hybrids demonstrated enhanced radical-scavenging capacity, superior thermal stability, and markedly reduced susceptibility to proteolytic enzymes, outperforming the unmodified protein. The assembly of Lf with food-grade polysaccharides yields a biocompatible delivery system that safeguards bioactive molecules through a combination of mechanical shielding, molecular stabilization, and gradual release of the bioactive molecules. Noteworthy progress has been achieved through the use of Lf–polyphenol conjugates that reinforce lipid emulsions without compromising protein activity, thus broadening the applicability of protein-polysaccharide interactions in emulsion technology [62]. Such naturally occurring complexation strategies confer a distinct advantage in the food industry, where the replacement of synthetic additives with renewable ingredients is often a regulatory and sensory objective, resulting in 2–3-fold enhancements in thermal and pH stability while preserving the inherent biological activity of the Lf. Gentle glycation of Lf generates stable Lf-lactose convergents that resist proteolytic attack while retaining approximately 70–85% of the protein’s original antimicrobial potency. Kinetic studies indicate that these resistant forms arise from an equilibrium between sliding equilibrium stoichiometries at 60 °C and 79% air moisture over 7–14 days, during which lactosyl and Lf moieties orient to react at strategic termini and core residue positions. The observed covalent crown shields vicinal peptide bonds from tryptic cleavage, extending the protein’s intractability in gastric and intestinal digesta. As detailed by Chen et al. (2022) [63], the thus-protected Lf retains 70–85% of its original bactericidal efficacy against *Salmonella*, *Listeria*, and enteropathic *Escherichia* strains, evidencing that regulated glycation can use glycar-powered polymeric interfaces to prolong both systemic longevity and gut-derived bioactivity.

### 5.3. In Vivo Strategies for Enhanced Bioavailability

The realization of successful in vivo protection mechanisms requires a thorough understanding of gastrointestinal function and the creation of systems that can adequately adjust to the wide-ranging conditions within the digestive system. Some recent breakthroughs in targeted release technologies include alginate composite and hybrid CaCO_3_-hydrogel beads designed for the oral delivery of Lf with the goal of intestinal targeting [58]. Sophisticated delivery systems provide more than 80% protection during the gastric phase and show controlled release behavior under intestinal conditions, leading to a 3–5-fold increase in bioavailability as opposed to unprotected Lf. The evolution of protective systems has been characterized by significant advancements, especially through the introduction of enteric microspheres and mucoadhesive devices that offer sustained protection via gastric retention. Chitosan microbeads constitute one of the most innovative methods of intensifying colon-specific drug delivery based on pH-sensitive swelling and release mechanisms that protect Lf from acidic environments but facilitate its release in the area of interest in the colon, where ideal drug effects can be attained [55]. Recent research suggests that it is possible to preserve the antibacterial activity of Lf when suitably formulated, even after exposing it to in vitro analysis of digestive processes that normally cause the denaturation of free proteins [59]. This finding has great promise for oral Lf supplementation and functional food product development, as specific formulation approaches could allow the maintenance of biological activity along the digestive tract. Bioenhancement methods encompass a range of techniques, including the use of natural permeation enhancers, synergistic compound co-administration, and synchronization of administration to achieve optimum efficacy. Novel formulations include the development of probiotic combinations to enhance Lf stability and bioavailability, the use of prebiotic synergism involving characteristic oligosaccharides to support beneficial bacterial growth and protect Lf, and the use of enzyme inhibitors to provide additional protection against gastrointestinal proteolytic degradation. The recognition of variability in gastrointestinal physiology and Lf metabolism has made it possible to design customized delivery systems that respond to individual needs based on age, adaptations related to specific diseases, and the effect of genetic factors on protein absorption and metabolism. Current research related to Lf focuses on the design of products to meet specific requirements at different life stages, optimizing the concentration for different developmental stages, designing disease-specific solutions for patients with compromised immune or gastrointestinal function, and using pharmacogenomics to enable personalized dosing according to an individual’s genotype [64].

## 6. Analytical Characterization After Isolation

An integrated analytical framework is introduced for the assessment of Lf following isolation, in which the characterization methods are intended to address distinct post-purification objectives. The analytical techniques are categorized in relation to their primary purpose: SDS-PAGE, Western blotting, and ELISA are implemented for verification of protein identity and purity, whereas circular dichroism (CD) and Fourier transform infrared (FTIR) spectroscopy are used to assess structural integrity and conformational preservation. In addition, functional assays are utilized to validate the retention of functional bioactivity. This organizational scheme highlights the requirement to select suitable characterization tools depending on the isolation methodology employed and the intended downstream application. Chromatographic and membrane-based isolation strategies principally aim to enhance yield and purity; nevertheless, their ultimate success is determined by the maintenance of structural attributes fundamental to iron binding and antimicrobial activity, which are directly or indirectly characterized through these analyses. Collectively, the analytical approaches outlined systematically correlate isolation outcomes with molecular integrity and functional performance, thereby providing a clear and rational basis for method selection and data interpretation.

### 6.1. Protein Identification and Verification

#### 6.1.1. SDS-PAGE

Sodium dodecyl sulfate polyacrylamide gel electrophoresis (SDS-PAGE) is widely used to evaluate protein purity and apparent molecular weight following Lf isolation. This technique enables verification of Lf enrichment and detection of co-purified protein contaminants, thereby providing a rapid assessment of isolation efficiency across different purification strategies [65]. As reported in the work of Abbas et al., 2015 [66], the purification efficiency was evaluated by performing denaturing SDS-PAGE following the Laemmli method. The protein profile of the isolated Lf appeared as a single distinct band and molecular weight was confirmed using a standard protein marker. The detection of a discrete band indicated the high purity of the isolated Lf and demonstrated the success of the Lf purification methods. A band close to 80 kDa was observed, although different molecular weight values have been reported due to variations in carbohydrate content [66]. As evidenced by previous studies, Chu et al., 1993 [67] determined the migratory mass of Lf isolated from porcine milk as 78 kDa, and SDS-PAGE was carried out to analyze the mass profile of the purified fraction. Following electrophoretic separation, silver staining was thereafter applied for protein visualization. An aliquot of the isolated Lf was loaded onto a resolving gel, and the molecular weight was confirmed, supporting the characterization of the purified Lf fraction [67]. In contrast, Elagamy et al., 1996 [68] analyzed Lf isolated from camel milk using SDS-PAGE and reported a relative mass of 79.5 kDa. The homogeneity of the isolated macromolecule was evaluated by SDS-PAGE analysis and the protein fractions were observed using Coomassie Brilliant Blue staining. The results supported the characterization of the isolated Lf based on molecular mass and purity [68].

#### 6.1.2. Western Blot

Western blotting (immunoblotting or protein blotting) was subsequently employed to enable specific detection of the target protein through antigen–antibody interaction. Upon completion of the separation of protein samples by SDS-PAGE, they were immobilized on nitrocellulose membranes using electroblotting and incubated with monoclonal primary antibody developed against bLf. This procedure was succeeded by exposure to a secondary antibody bound to horseradish peroxidase (HRP). Upon binding to the primary antibody, the HRP enzyme facilitated the detection of the Lf–antibody complex via a chemiluminescent reaction. Hence, both the presence and the relative quantity of the target protein were confirmed. The current study aimed to investigate three different commercial bLf samples that were analyzed: mature milk Lf (mbLf), colostrum Lf (cbLf), and whey-based colostrum (wbLf). SDS-PAGE analysis of mbLf demonstrated a solitary band corresponding to ~80 kDa, indicating high purity and structural integrity of the protein. In the cbLf sample, two migration zones were identified at ~80 kDa and ~45 kDa; the latter was interpreted as an indication of possible contamination or protein degradation. In the wbLf sample, the appearance of multiple bands around 80 kDa suggested the presence of other proteins, particularly lactoperoxidase. Moreover, the differences in molecular weight between the mbLf and cbLf samples were attributed to isoform variations and post-translational modifications, particularly glycosylation. Through the combined application of SDS-PAGE and Western blotting, the presence of intact bLf protein was specifically verified. Even though multiple bands were observed in SDS-PAGE due to possible contamination, degradation, or the presence of unrelated proteins, the appearance of a single band on the membrane after Western blotting with anti-bLf antibodies confirmed both the identity of the protein and the success of the purification protocol [69,70].

#### 6.1.3. ELISA

Enzyme-linked immunosorbent assay (ELISA) is a serological test that works by interacting with an immunoreactants, namely a primary antibody directed toward the antigen (i.e., the analyte protein). The presence of the analyte is detected by the addition of a reporter antibody to the detection reagent, yielding a readout that is quantified spectrophotometrically or qualitatively using a luminometer. ELISA methods vary according to the type of interactions, how the signal is derived, and assay specificity; these include direct, indirect, sandwich, and competitive ELISA. Nonetheless, they have different classifications, they all fundamentally depend on antigens, antibodies and substrates. These formats enable detection and quantitative evaluation of the target protein following isolation procedures [71]. Evidence presented by Ostertag et al. 2022 [72] indicates that the structural and functional integrity of Lf, along with its content during various dairy processing steps, was tracked using competitive ELISA method. Lf concentration levels were assessed based on their capacity to discriminate relative to output amplitudes. The limit of detection (LOD) was set as the minimal quantity that could be discriminated from the blank, with measurable samples containing >3.9 ng per mL Lf. Furthermore, the limits of quantification (LOQ), defined as the minimal and maximum thresholds over which quantification is feasible, were reported as 3.9–250 ng per mL for LOQ (lower) and 600–2000 ng per mL for LOQ (upper). Notably, the natural Lf concentration in skimmed milk was found to exceed the LOQ (upper) value by approximately 200-fold. In conclusion, quantitative measurements performed by competitive ELISA revealed recovery rates in dairy-derived formulations including whey (107%), cream (106%), milk (104%), and curd (96%) [72].

### 6.2. Analysis of Structural Identity

#### 6.2.1. Circular Dichroism (CD)

Circular Dichroism (CD) is considered to be differential capture of light depending on its polarization state as it passes through molecules. 190–250 nm is the spectral region in which the folding patterns in polypeptide are capable of being characterized by CD spectroscopy, as the chromophore in this range is the amide linkage, and the spectral feature becomes pronounced in a well-folded and ordered environment. Secondary structure elements, whose representative forms include α-helix, β-sheet, or random coil, exhibit characteristic shapes and intensities in the CD spectrum. Additionally, 250–320 nm spectral region is indicative of specific attributes of a protein’s higher-order folding. Within this spectral interval, the primary chromophores are aromatic side chains and disulfide bridges, whose CD signals reflect subtle modifications in tertiary structure resulting from interprotein associations or variations in solvent composition. For instance, following a chemical or structural modification on a protein, CD spectroscopy allows for comparative analysis between the modified and native forms. Furthermore, by monitoring the CD signal at a single wavelength corresponding to a specific secondary structure element, namely the α-helix, a thermal unfolding profile can be generated by incrementally increasing the temperature [73]. An experimental study by Álvarez-Mayorga et al., 2024 [74] demonstrated how the structural integrity of recombinant human lactoferrin (rhLf) and native human lactoferrin (nhLf) is impacted by changes in pH and temperature using CD spectroscopy. At pH 7.0, the CD spectral pattern of rhLf exhibited two negative peaks at 208 nm and 220 nm, indicating a chiefly α-helical architecture. On the other hand, as the pH increased, both signals showed a decrease in mean residual ellipticity (MRE) values, which is indicative of a denaturation process. Conversely, when the pH was lowered, a decrease in all MRE values was observed, which was attributed to the formation of precipitates as a result of reduced amount of soluble protein. Even though this technique is not intended for direct quantification of iron-binding capacity, it yields insight into the structural integrity of the protein through assessments grounded in secondary structure parameters. In this context, comparison across distinct physicochemical conditions reveals the circumstances under which Lf maintains its native conformation, thereby enabling the characterization of an indirect structure–function correlation linking the structural state of iron-binding regions with their functional retention [74]. Another study illustrating that conformational changes can be evaluated by CD spectrometry under varying pH conditions was conducted by Sreedhara et al., 2010 [75]. In this work, caprine and bLf were analyzed at pH values of 2.0, 5.0, and 8.0. It was reported that the secondary structure of bLf remained largely preserved within the pH range of 5.0 to 8.0, with only minor architectural changes observed [75].

#### 6.2.2. (Fourier Transform Infrared) FTIR

Fourier Transform Infrared (FTIR) spectroscopy yields information about the higher-order structures of proteins through the application of infrared radiation to the analyte and observing how it interacts with radiation at light intervals in the infrared range of the spectrum. Protein vibrational bands corresponding to Amide I and Amide II, which constitute the signature features in the infrared spectra, are widely analyzed. The Amide I band is correlated with absorption arising from the stretching vibrations of the C=O bond, whereas the Amide II band corresponds to the bending vibrations of the N–H bond. Analysis of these bands using an FTIR spectrometer allows for in-depth insight into the secondary structures of proteins [76]. The work of Pryshchepa et al., 2022 [77] revealed that the FTIR spectra peak profiles of native bovine lactoferrin (nbLf) and Fe^3+^ ion-interacted bLf were compared. Distinct spectral changes between nbLf and iron-loaded bLf were examined. As a result of the binding, changes in specific functional groups and chemical bonds were assessed in comparison with the native form. Fe^3+^ ion binding led to band weakening accompanied by red shifts, which was assessed in relation to the interaction between the C–H band and metal cation binding. After iron addition, shifts in these bands were recorded. This structural variation can be interpreted as resulting from iron binding–induced reshaping of the secondary interaction network within the protein. Whereas FTIR spectroscopy is not primarily intended for direct quantification of iron-binding capacity, it offers insight into changes in secondary structural integrity through amide bond vibrations and the associated maintenance of functional properties. The manifestation of these secondary structural alterations as band shifts in FTIR spectra implies that iron-binding domains require a properly folded structure to remain functionally active. The capability to monitor this conformational integrity via FTIR therefore constitutes an indirect yet robust structural quality assessment tool for isolation and purification workflows. In conclusion, the secondary structural features of the protein during Fe^3+^ ion binding to bLf were characterized, the analysis yielded valuable information about the protein’s structural integrity [77].

### 6.3. Functional Assays

In 2016, Redwan et al., 2016 [78] undertook an in-depth examination aimed at evaluating the minimum inhibitory concentration (MIC) assay of Lfs purified from camel (cLf) and hLf milk that effectively impede the propagation of methicillin-resistant *Staphylococcus aureus* (MRSA) and methicillin-sensitive *Staphylococcus aureus* (MSSA). MRSA and MSSA bacteria were standardized to 2 × 10^6^ CFU/mL and incubated in media containing different Lf concentrations (0, 0.25, 0.5, 0.75, 1, 1.5, 2, 2.5, and 3 mg/mL), and microbial proliferation was monitored optical density (OD) at 620 nm to determine MIC values. An agar disk diffusion test was executed to analyze the susceptibility of MRSA and MSSA to cLf, hLf, and 17 diverse antimicrobial agents on Mueller-Hinton agar. The cLf concentration fluctuated between 1.95 and 2 mg/mL, whereas the hLf concentration spanned 3 to 3.9 mg/mL; these intervals were defined based on MIC values obtained from antibiotic-containing media. Results indicated that Lfs exhibited synergistic effects with vancomycin and oxacillin, while fusidic acid showed an indifferent interaction. A time-kill assay was additionally employed to evaluate the antimicrobial kinetics of cLf and hLf alone and in combination with antibiotics. Given Lf’s known iron-chelating property, the relationship between antimicrobial activity and iron saturation was investigated. Despite its higher iron saturation, cLf demonstrated stronger antimicrobial activity against MRSA. These findings indicate that while iron chelation contributes to Lf’s antimicrobial effect and structural differences between cLf and hLf, including N-terminal regions and glycosylation patterns, may also play a role [78].

## 7. Optimization Strategies

Optimization strategies emphasize preserving the structural and functional integrity of Lf after isolation rather than its initial retrieval from raw matrices. In addition to assessing pH, temperature, and processing settings as operational determinants for high-performance fractionation, these parameters are analyzed within an optimization framework regarding their impacts on molecular stability, iron-binding capacity, and bioactivity. Consequently, pH, temperature, and processing time are evaluated as key drivers of conformational stability and functional preservation. In parallel, the use of protease inhibitors and stabilizing agents is addressed as a strategy to minimize degradation and depletion of biological activity throughout processing and storage. Finally, protein engineering approaches are considered in the context of maintaining molecular fidelity and operational performance in recombinant Lf expression systems. Collectively, these strategies establish a framework for optimizing lactoferrin quality exceeding conventional yield and purity metrics.

### 7.1. Effects of pH, Temperature and Time

Lf stabilizes by means of hydrogen bonds, hydrophobic/hydrophilic interactions, ligand binding, and disulfide bonds in its molecular framework. In view of the fact that changes in pH, temperature, and time exert a direct impact on these forces, they consequently culminate in destabilization; for this reason, in related studies, proper optimization of these environmental factors is pivotal. pH exerts control over salt bridges and hydrogen bonds, given that the medium’s pH is within the isoelectric point (pI) range of 8.0–9.0, and any deviation from this interval affects these interactions. The phenomenon emerges when the pH of culture deviates from the pI point, Lf becomes soluble in water, which in turn alters solubility rates. Indeed, in a study investigating solubility, it was concluded that at pH 7, Lf exhibited over 92% solubility. Furthermore, it was found that in the pH range of 5.0–6.5, Lf begins to liberate iron, and at pH 2, more than 90% of the iron is released, leading to the apo-form, which subsequently affects its thermal stability; following this, Lf was observed to gel at neutral and alkaline pH values, whereas it remained stable at pH 4. Variations in thermodynamic state have been indicated to modulate the kinetic energy associated with hydrogen bonds, as well as damage hydrophobic and thiol/disulfide interactions. In a study evaluating the denaturation of Lf with respect to its structure, purified apo-Lf was detected to denature at approximately 70 °C, whereas purified holo-Lf structurally altered at around 90 °C and at pH 6.0–7.0. Accordingly, it has been validated that the iron-bound form manifests superior resistance to denaturation at heightened temperatures as saturation increases. To better understand the native structure and denaturation process of Lf, studies have also been conducted using various temperature treatments, among which are pasteurization, sterilization, and ultra-high temperature (UHT) processing. Several types of milk tested, among which bovine, buffalo, and camel milk samples at their natural pH were first subjected to 65 °C for 30 min without substantial denaturation observed; even so, exposure to 85 °C for 30 min resulted in denaturation accompanied by changes in biological activity. Comparative studies over time have been conducted by heating bLf at distinct heating times under increased thermal conditions. Initially, bLf was heated at 70 °C for 3 min, succeeded by exposure to UHT treatment at 130 °C for 2 s under pH 4.0 conditions, resulting in no detectable impairment of Fe-chelating potential. Nonetheless, when bLf was subjected to 120 °C for 15 min (pH 2.0), complete loss of iron-binding ability was reported; consequently, exposure time was indicated to substantially affect Lf’s biological activity [5]. Denaturation and functional losses also fluctuate with time at subzero temperatures, with different preservation criteria. In a study conducted on hLf, it was reported to remain stable between −18 and −20 °C for 5 days, yet underwent 37% denaturation after 3 months and 46% after 6 months attributable to progressive changes over time [79].

### 7.2. Use of Protease Inhibitors and Stabilizers

Protease inhibitors in milk (Lf, β-casein, cystatins) contribute significantly not only in sustaining milk quality but also in defending against microorganisms. Therefore, in isolation studies, preserving the structural integrity of these proteins and accurately fractionating their activities is of paramount relevance. Varying Lf contents from diverse origins acquire importance due to the quantity and efficacy of protease inhibitors during isolation, as colostrum exhibited around two- to three-fold more Lf compared with mature milk. Since stronger defense against proteases leads to a more enhanced isolation process. One of the methods used to investigate the presence of protease inhibitors is reverse zymography. In this approach, proteins within the sample are resolved by SDS-PAGE after electrophoresis, afterwards SDS is removed as SDS inhibits protease activity. Subsequently, the gel is immersed in a protease solution, such as one containing papain. If there are protease inhibitors within the bands formed on the gel, papain activity is inhibited in those regions; thus, after staining with Coomassie Brilliant Blue, the respective regions remain stained. This occurs because the gel has not been degraded, appearing as blue bands, indicating the presence of protease inhibitors in the sample [80]. In an analysis undertaken by Richards et al., 2014 [81], the suppression of the activities of the natural milk protease plasmin, as well as bacterial proteases from *Bacillus* spp. and *Pseudomonas fluorescens*, was assessed by means of legume-derived protease inhibitors. The data indicated that the legume protease inhibitors inhibited *Bacillus* spp. serine proteases by over 90%, whereas their inhibitory impact on *Pseudomonas fluorescens* proteases was confined to 4% to 19%. The study also highlighted that, in addition to the involvement of phenolic constituents detected in the fractions, which form complexes with proteases and enhancing inhibition, legume protease inhibitors demonstrate specificity towards particular protease types. Therefore, it was concluded that careful selection of protease inhibitors is fundamental to related studies [81]. Evidence from Xiong et al., 2021 [82] indicates that the impact of additional milk proteins and stabilizers on Lf aggregation were evaluated. The occurrence among dairy-derived polypeptides namely β-lactoglobulin (β-LG) was documented to be instrumental in the heat- and pH-induced denaturation of Lf and to maintain its structural integrity throughout the denaturation process. In thermal treatments were performed at 65 °C, 70 °C, and 75 °C for 30 min with Lf and β-LG, an rise in turbidity, signifying intensified aggregation, was recorded with increasing temperature, and disulfide-linked aggregates of Lf were identified in the heated samples. The aggregates generated revealed the conformational alterations in Lf. While aggregation in Lf heated without milk proteins began at 65 °C, when heated in combination with milk proteins, aggregation was recorded at 75 °C and beyond, demonstrating that milk proteins create a protective layer on the exterior of Lf and retard its denaturation. The disaccharide stabilizers sucrose and trehalose preserved the biological activity of Lf while also minimizing significant modifications in its biofunctional characteristics under pH and temperature variations. Trehalose, known as a potent water-binding agent, associates with water molecules to maintain the hydration layer on the surface of Lf, thereby increasing its structural flexibility and preventing denaturation in this context [82].

### 7.3. Protein Engineering (Structural Preservation in Recombinant Systems)

Biopharming systems leveraging genetically modified organisms for the synthesis of therapeutic proteins provides the means for attainment of Lf with substantially higher quality, stability, and purity compared to standard sources, owing to the regulated production setting [83]. A diverse range of viable sources for the production of pure Lf. The first is the prokaryotic system of *Escherichia coli* (bacteria), in which the likelihood of contamination is minimized. A multitude of Lf variants have been expressed employing this system. One example is the buffalo N-lobe Lf expressed using the pQE30 vector in the *E. coli* B21 (DE3) strain, generating an Lf concentration of 1 mg/mL [84]. As an additional Lf source, hLf was generated through the pET28a+ vector, reaching a concentration of 2.9 mg/mL [85]. Functioning as an expression host, the eukaryotic yeast *Komagataella phaffii* (formerly known as *Pichia pastoris*) permitted the secretion of heterologous protein employing high-density fermentation. Using the *P. pastoris* GS115 strain, porcine Lf (pLf) was produced in an amount of close to 2.8 g/L [86]. In addition, production of the cationic peptide obtained from Lf was executed employing the same strain, resulting in 11.5 mg/L of Lactoferricin B (LfcinB) [87]. In filamentous fungi (molds), which are utilized for Lf production because of their high yield and proper protein folding, hLf was manufactured via Aspergillus oryzae using the pAhLFg vector, yielding 25 mg/L [88]. Owing to restrictions in accessing bioreactors, the synthesis of hLf recombinantly via transgenic cows has been adopted as an alternative to transgenic animals. Despite the fact that hLf was produced at gram-per-liter concentrations, certain discrepancies were detected between rhLf and natural hLf. Chief among these differences was post-translational sugar modifications, with the sugar structure of rhLf found to differ from that of hLf. Transgenic cow-derived Lfs, which attained prominence for their biopharmaceutical roles, were tested in both normal (immunocompetent) and immunosuppressed mice. The results showed that rhLf exhibited biological activity and infection defense equivalent to natural hLf [89]. In a study focusing on ligand-receptor interaction in recombinant Lf production, cloned human Lf was synthesized in hamster kidney cells. For the receptor binding competition assay, three different forms of rhLf were created: N-lobe alone, N-lobe with glycans removed by PNGase, and a deliberately introduced N137A mutation (N-lobe with modified glycosylation site). Data obtained from the assay interrogated by laser-mediated desorption/ion generation coupled with time-of-flight mass spectrometric measurement, demonstrated that although receptor binding affinity was diminished, the N-lobe of Lf still retained the glycans necessary for receptor binding [90]. While investigations focused on rapidly achieving target characteristics in protein engineering continue, labor-intensive methods are progressively being succeeded by innovative procedures. One such study is the novel PCR-based mutagenesis method called Direct, conducted by Watanabe et al., 2021 [91]. This strategy permits over 99% high-fidelity mutations, and by means of intracellular protein biosynthesis, the prevalence of recombinant DNA technologies is diminishing or shifting directions. The execution of this theory was on a key enzyme, NADPH-dependent 3-quinuclidinone reductase from *Rhodotorula rubra.* Not only were 90 new mutant proteins obtained in a single screening cycle, but also a mutant with optimized properties (Q135I) was isolated [91].

## 8. Applications and Relevance

### 8.1. Pharmaceutical and Biotechnological Applications

In light of the development of drug-resilient microorganisms, individuals are resorting to non-conventional treatments extracted from bio-derived sources instead of conventional antimicrobial drugs. Due to the fact that Lf serves as an antimicrobial agent, it has provided a novel resource for prospective pharmaceutical opportunities. In addition, segments or variants of Lf, such as Lf (1–11), lactoferricin (Lfcin), and lactoferrampin are as potent as the intact protein [92]. Among the reported studies exploiting the antiviral property of Lf was conducted on chickens against bursal disease (IBD) virus, in which recombinant porcine lactoferrin (rpLf) was administered. In essence, rpLf functioned to strengthen immunological activity [93]. Investigations have proposed that Lf initiates the host’s learned and inherited defense mechanisms [94]. In the course of immunomodulation, Lf is synthesized by immune cells and acts as an alarm signal [95]. Given that Lf regulates the modulation of immune responses under pathophysiological circumstances, research efforts have corroborated its protective impacts in several inflammatory conditions [96]. Lf also confers the capability to control cytokine biosynthesis in cancer [97,98]. In an investigation of the anti-tumor property of Lf, the enteral delivery of bLf to murine models has been validated to markedly suppress tumor formation and exhibited anticarcinogenic activity. Similarly, rhLf administered to mice inhibits tumor growth [99,100]. Consequently, the application of Lf has increased the effectiveness of innate immune defense mechanisms and facilitated to the prevention of pathogen invasion. According to findings, supplementation with Lf resulted in increased immune stimulation [101]. Isolation of Lf from native sources is suboptimal for commercial-scale therapeutic utilizations; therefore, recombinant protein expression systems serve as a strategy for the high-yield production of Lf. Throughout the application of expression systems, purification methods and optimization strategies are key factors in biotechnological applications. One of the key considerations in these studies is achieving biologically active Lf; therefore, the strategies chosen during production demand a well-optimized and multidisciplinary approach, involving analytical characterization [102].

### 8.2. Importance of Functional Protein in Clinical Research

By virtue of its potential in the treatment of a broad spectrum of disorders, Lf is widely employed in clinical investigations; nevertheless, its bioactive conformation must be preserved prior to the intended application. Thus, an analytical approach should be employed for stability [103]. Lf, owing to its three-dimensional structure, interacts with a broad array of receptors, thereby demonstrating considerable effectiveness in the resolution of pathologies [104]. Research has explored the inhibitory effect of this cationic immune protein on biofilm establishment by Pseudomonas aeruginosa and Streptococcus mutans. Lf suppressed biofilm formation in *Pseudomonas aeruginosa*, whereas in *Streptococcus mutans* iron deprivation stimulated biofilm formation, validating pathogen-specificity in clinical applications [105]. Based on findings presented by Manzoni 2016 [106], the contribution of breast milk-derived factors to neonatal host defense was highlighted. Lf demonstrates a high stability following oral administration, which is attributed to its resistance to pepsin-mediated cleavage and the subsequent release of lactoferricin [106]. In humans, the Lf content differs in mature milk. Findings indicate a mean concentration of 7.0 ± 5.1 mg/mL in term infants, while it is 2.3 ± 0.4 mg/mL in mature milk [107]. Lf exerts a mucosal trophic effect by influencing intestinal permeability in infants. It serves as regulator of intestinal permeability [108]. Compared to formula feeding, breastfeeding facilitates maturation of the intestinal epithelium [109]. Fecal Lf concentrations increased from birth to 1 month in both term and preterm infants [107]. Furthermore, the trophic effect of bLf and hLf on intestinal function development was determined to be concentration dependent. Although no significant difference in intestinal *Candida* colonization was observed, Lf mitigates infection by preventing *Candida* translocation and strengthening the intestinal barrier [110,111]. An additional therapeutic effect of Lf has been evaluated in female long-distance runners prone to anemia arising from insufficient iron. These findings reveal that Lf supplementation may enhance iron bioavailability and incorporation [112].

### 8.3. Use in Nutritional Supplements and Functional Foods

Studies have demonstrated that many newborns cannot be breastfed; therefore, the production and application of infant formula has become inevitable. In this context, Lf is of notable relevance and fortification of infant formula with Lf is feasible through large-scale industrial production of bLf from skimmed bovine milk and whey. Since the 1990s, bLf has been added as a supplement to commercial infant milk in Japan. To facilitate such an application, bLf was granted “Generally Recognized as Safe” (GRAS) status by the U.S. Food and Drug Administration (FDA) in 2001, and was subsequently sanctioned as a food additive. Furthermore, in 2012, the European Food Safety Authority (EFSA) approved it to serve as an innovative dietary component and Lf has since been incorporated into nutraceutical supplemented infant formula and various foodstuffs [113,114,115]. Only 1% Lf content was quantified in the feces of infants six weeks after birth, indicating high bioavailability of milk-derived proteins [116]. Therefore, farmers, producers, and researchers consider Lf, described as a “miracle” molecule, as a dietary supplement that, when administered in appropriate doses, can boost animal production and performance while also mitigating reliance on antibiotics [117]. Lf, as part of the whey protein fraction, fails to integrate into the curd during cheese production; however, Lf-fortified Cheddar cheese was formulated, indicating that Lf may serve as a supplement in cheese manufacturing [118]. Sharma et al. reported that oral administration of milk bio-processed using *Lactobacillus fermentum* MTCC 5898 (LF) resulted in improved immune-related functions in aged mice, supporting the employment of Lf as a nutritional adjunct [119]. In an investigation into the application of natural antimicrobials for food preservation, the applicability of activated Lf (ALF) and rosemary extract (RE) against *E. coli* O157:H7, *Salmonella Enteritidis*, and *Listeria monocytogenes*. Furthermore, the integrative action of ALF and RE on *E. coli* O157:H7 inhibition resulted in an augmentation of the activity of ALF by two- to three-fold [120]. According to the investigation of Brown et al., Lf was incorporated into chitosan, and the efficacy of a natural protective coating antagonistic to food-associated microorganisms was demonstrated, indicating a synergistic effect between the two components [121].

## 9. Conclusions

Considering its molecular configuration, Lf, described as a “miraculous molecule,” has broadened its applicability, elevating production rates each year. Its capacity to interact synergistically with other bioactive components is only one of the criteria for this expanding range. Being a natural compound, its functionality has made it prominent across multiple sectors. As an illustration, its use in functional foods allows direct utilization by consumers. Moreover, its application as packaging material eliminates concerns regarding food hygiene, by sustaining robust protection against even the most hazardous foodborne pathogens. With this unique property, Lf persists in shaping the processes of isolation, purification, and optimization rapidly. To meet the growing demand in its applications, the isolation of highly pure and active Lf from sources has also achieved widespread adoption. This popularity has permitted the advancement of technologies with reduced processing durations and high efficiency purification strategies. Prior to these advanced isolation technologies, the fundamental phase is establishing optimal physicochemical conditions to optimize the process. Parameters including pH, temperature, degradation, denaturation, and protease activity must be optimized to guarantee that the isolated protein preserves its bioactive structure throughout subsequent processes. In the course of optimization, the protein’s ionic characteristics are determined based on its pI point for setting the ideal pH range, and the binding and elution conditions in ion-exchange chromatography are adjusted accordingly. With the expanding applications of Lf, its recombinant production via protein engineering using transgenic animals targets high-yield production. As biotechnological applications aim to scale industrially, both the models used, and the applied methods must be continuously updated to meet the growing future demand.

## Figures and Tables

**Table 1:** Detailed research studies regarding the chromatographic methods.

Method	Lf Origin	Initial Matrix	Purification	Additional Processing	Analysis and Validation	Lf Yield (%)	Purity (%)	Advantages	Disadvantages	Reference
Affinity Chromatography	Bovine (transgenic milk, rhLf)	Skim milk whey	Affinity Chromatography (heparin sepharose)	- Precipitation with HCL - Ion-exchange chromatography (high Q) Dialysis 48 h, MWCO 12–14 kDa	- SDS-PAGE - Bradford Assay	NR	NR	- High initial rhLf concentration - Preserved protein stability - Lf fractions suitable for N-glycan analysis, indicating preservation of structural integrity	- Yield and purity not reported - Biological activity of some fractions requires further validation - Functional activity after isolation was not directly evaluated.	[41]
	Goat	Goat milk	Affinity Chromatography (polypropylene column)	- Precipitation with HCL and CaCl_2_ - 50 kDa cut-off centrifugal filter device (Amicon, Millipore, Billerica, MA, USA)	- %12 SDS-PAGE - Bradford Assay	NR	NR	- Precipitation with Ca^2+^ eliminates casein micelles	- Lf relatively low levels in goat milk - High concentrations of casein can contaminate Lf - Structural integrity and functional activity were not directly assessed after isolation.	[42]
	Human	Human colostral whey	Affinity Chromatography (immobilized ssDNA-agarose)	- High-performance ion-exchange (Mono-S) - Cu^2+^-immobilized metal ion affinity chromatography (IMAC) - Reverse-phase C18 chromatography	- SDS-PAGE (silver staining) - UV-VIS spectroscopy - ^59^Fe-binding assay (analyzed by HPLC size-exclusion TSK-3000SW and Mono-S ion-exchange) - Ouchterlony immunodiffusion (with anti-hLF antisera, Dako Laboratories (Santa Barbara, CA, USA)) - Western blot (peroxidase-conjugated secondary antibody)	>95%	High purity (silver stain)	- One-step purification with high yield - Applicable to both apo- and holo-LF - Confirm homogeneity under different physicochemical properties - Rapid, non-destructive determination of iron saturation - Demonstrates functional activity of LF - Confirms antigenic integrity and immunological specificity	- Extra cost and equipment; time-consuming; not essential for routine purification - More laborious and costly than Coomassie; less quantitative - Only shows iron-binding status; does not confirm purity - Use of radioactive isotope requires safety precautions and ethical considerations - Functional activity was not directly assessed after isolation.	[43]
	Human	Human whey	Immobilized Metal Affinity Chromatography (IMAC) using Cu(II)-IDA-agarose	Casein precipitation with acetic acid (pH 4.6)	SDS-PAGE (Coomassie Brilliant Blue, analytical grade)	NR	NR	- High selectivity for histidine-rich proteins - Reusable column without significant loss of capacity - Relatively fast and efficient purification	- Requires pH/imidazole elution steps - High cost of resin - Structural integrity and functional activity were not directly assessed after isolation.	[44]
Ion-Exchange Chromatography	Bovine	Raw whole milk	Strong Cation Exchange Chromatography (SP sepharose big beads)	NR	- ELİSA - Surface Plasmon Resonance (SPR) - (goat-polyclonal anti-bovine Lf) - BCA Protein Assay	~95% (leakage 4.6%)	NR	- High binding capacity (~48 mg/mL resin) - Simultaneous Purification (LF and LPO) - Preservation of milk components - Reusable column	- Resin capacity depends on solution concentration - Equipment is costly - Optimization required for temperature and flow rate - Structural integrity and functional activity were not directly assessed after isolation.	[45]
	Bovine	Whey	Cation Exchange Chromatography (SP Sepharose FF colon, pH = 6.7)	NR	- SDS-PAGE - Design of Experiment (DOE) - Response Surface Modeling (RSM) - Mechanistic Model (SMA)	NR	~95%	- LF separated from LPO with high selectivity - High purity achieved - Modeling facilitated optimization - Two value proteins purified in a single step	- Yield percentage not reported - Costly at industrial scale - Structural integrity and functional activity were not directly assessed after isolation.	[46]
	Bovine	Crude sweet whey (cheese-by product, unprocessed)	Cation Exchange Expanded Bed Chromatography (Resin = Fastline SP, pH = 7.0)	NR	- SDS-PAGE - ELİSA - BCA Protein Assay - Kjeldahl nitrogen method - K2 600 spectrophotometer (280 nm) (Knauer, Berlin, Germany) - Ultrafiltration (10 kDa cut-off)	77.1%	88.5%	- Expanded bed chromatography is suitable for whey - Requires no pre-treatment - Achieves a high purification factor (533) in a single step	- The obtained elution volume is large (268 mL) - Requiring concentration - Equipment and resin are costly - Structural integrity and functional activity were not directly assessed after isolation.	[47]
	Bovine	Sweet whey	Cation Exchange Expanded Bed Chromatography	- Microfiltration (cellulose membrane 0.22 µm) - Ultrafiltration (polyethersulfone membrane of 30 kDa) - Diafiltration (6 diavolumes, 30 kDa PES membrane)	- Bradford assay - HPLC (Reverse phase, C18 apHera) - Diode array detector (DAD) (280 nm)	87%	92.7%	- Column capacity optimized by increasing concentration through ultrafiltration - Process more efficient than conventional methods - Integrated MF–UF–DF–EBC process enabled efficient purification	- Adsorption capacity decreased at higher pH and salt - High resin and equipment costs - Very low initial LF concentration in sweet whey - Structural integrity and functional activity were not directly assessed after isolation	[48]
	Bovine	Colostrum	Strong Cation Exchange Chromatography (fast flow, preperative scale)	- Casein precipitation with HCL (pH = 4.2) - Two step ultrafiltration; UF1: 100 kDa UF2: 10 kDa	- SDS-PAGE - ELİSA (mouse anti-Lf) - HPSEC (Agilent 1100 HPLC, Zorbax GF-250 column) (Agilent Technologies, Palo Alto, CA, USA)	94.04% (UF-2 retentate) 82.46% (Final purified Lf)	30.88% (UF-2 retentate) 94.20% (Final purified Lf)	- Combination of UF and strong cation exchange chromatography yielded high purity and good recovery - Reported to be more efficient compared to conventional commercial methods.	- UF operation is sensitive to transmembrane pressure (TMP) and flow velocity, requiring optimization - Purification process is multi-step and operationally complex - Advanced equipment and membrane costs may be high - Structural integrity and functional activity were not directly assessed after isolation.	[17]

NR: Not Reported.

## Data Availability

No new data were created or analyzed in this study. Data sharing is not applicable.
